# Space-Time-Stratified Case-Crossover Design in Environmental Epidemiology Study

**DOI:** 10.34133/2021/9870798

**Published:** 2021-10-07

**Authors:** Yao Wu, Shanshan Li, Yuming Guo

**Affiliations:** School of Public Health and Preventive Medicine, Monash University, Melbourne, VIC, Australia

We are living in a changing environment that affects human health. It is vital to use proper methods to quantify the impact of environmental exposure (e.g., air pollutants and extreme temperatures) on human health. Case-crossover design with daily environmental exposure and health outcomes (e.g., deaths and hospitalisations) is one of the most common study designs. It allows researchers to examine the acute health effects due to short-term environmental exposure.

A case-crossover design utilizes the ID as a stratum, comparing individuals to themselves at different times. To examine whether the events are associated with a particular exposure, it compares exposure level in the day when the health event occurs (case day) with the levels in nearby days (control days). The control days represent the counterfactual exposure experience of each case, independently of the exposure on case day. With this design, the long-term trend and seasonality of unmeasured variables are controlled for [2]. Several strategies for choosing control days are proposed (Figures 1(a)– 1(e)) [3, 4]. However, unidirectional and bidirectional strategies introduce biases from various sources, such as time trends and seasonal patterns in exposure or health events and nonindependent selection of control days [5]. Time-stratified case-crossover design is the best approach to control the above biases. 

**Figure 1:**
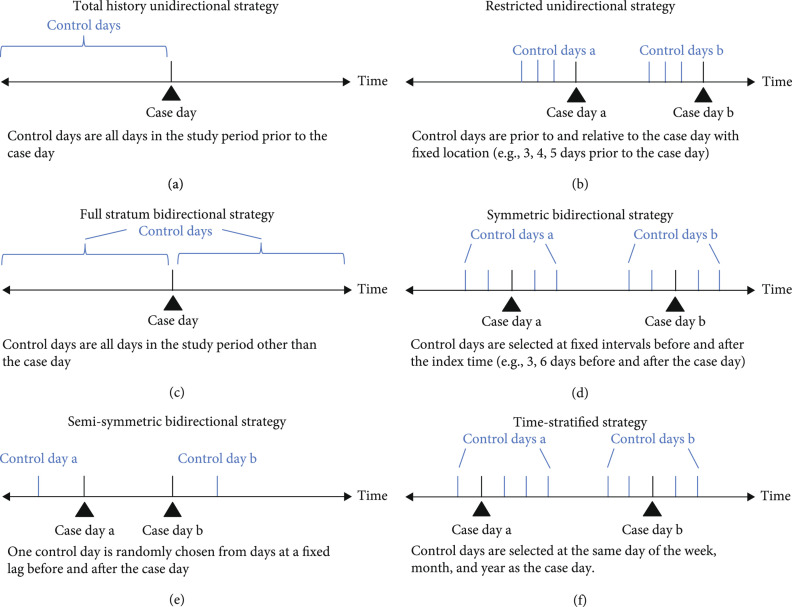
Strategies of control day selection in the case-crossover study.

The time-stratified case-crossover design has been widely used for location-specific time-series data [6]. Recently, multilocation time-series data are used to examine the health impacts of environmental exposures, to make the results generalizable, credible, and confirmable. To accommodate the increasing demand for multilocation studies, we propose the space-time-stratified case-crossover design which is developed from the time-stratified case-crossover design and applied to multi-time-series data. This method is characterized by the fact that each individual serves as her or his own control. For each case, it introduces a stratum combining two dimensions of time (e.g., month) and space (e.g., study locations). Specifically, within each ID stratum, the case day and control days are matched by day of the week in the same month, in the same year, and in the same location (e.g., city). Thus, each case has 3 or 4 control days (before and/or after the case day in the same month, Figure 1(f)). This design allows researchers to simultaneously control for the impacts of the day of the week, seasonality, long-term trend, and spatial variation using location-specific fixed and disjointed time strata and to avoid bias resulting from time trends by removing patterns in the placement of referents [7, 8]. In addition, it can also adjust individual characteristics which are unlikely to change within the small-time window, such as demographic characteristics (e.g., sex, race, education, and weight) and living habits (e.g., smoking and drinking). 

Two statistical models, conditional logistic regression and conditional Poisson regression, can be used to perform the space-time-stratified case-crossover study. Conditional logistic regression is similar to the analysis of matched case-control study, which requires the dataset to be expanded from time-series format to individual matched case-control format [3]. For every case occurring on a day, the day of the case is defined as a “case” and other days in the same stratum (on the same day of the week, month, year, and the same location) as “controls.” If there are k cases in the day i, there must be k stratums of the day i in the data set. Variables indicating the long-term trend and seasonality are not necessary to be included in the model. The equation is as follows:

(1)logitPcase=1instratumi∣expo,covariates=astratumi+β0×expo+βT×covariates,

where a stratum consists of 1 case (case=1) and its 3 or 4 controls (case=0), $P\left(\text{case}=1\text{in}\text{stratum}i|\text{exposure},\text{covariates}\right)$ is the conditional probability of being a case in the i^th^ stratum given the value of exposure variable and other covariates, $a_{\text{stratum}i}$ represents the constant or intercept of stratum i, expo stands for the exposure variable of interest in the study with its coefficient $\beta_{0}$, covariates stand for variables adjusted in the model, and $\beta^{T}$ denotes the coefficients of covariates. 

On the contrary, a conditional Poisson regression model can be performed directly on data with a time-series format (a sequence of daily cases indexed in time order), to fit space-time-stratified case-crossover design. This means the aggregated count data instead of individual data are needed [9]. Under its design, conditional Poisson (quasi-Poisson) regression allows researchers to adjust for overdispersion and autocorrelation in the count data, which is not possible for conditional logistic regression. The equation is as follows:

(2)LogEY=a+β0×expo+β1×stratum+βT×covariates,

where $E\left(Y\right)$ stands for the expectation of daily cases, a is the intercept, stratum is the location-specific time window defined by researchers by grouping the same day of the week within each month of each year in the same location, expo stands for the exposure variable of interest in the study with its coefficient $\beta_{0}$, covariates stands for variables adjusted in the model, and $\beta^{T}$ denotes the coefficients of covariates. 

We give examples of the application of space-time-stratified case-crossover design to examine the association between diurnal temperature range (DTR) and death counts. Data from a previous study was used, which contains daily death counts from 10 regions of England and Wales [10]. The stratum is defined as a categorical variable of the region-specific year, month, and day of the week (e.g., region York&Hum-1993-January-Friday). The nonlinear relationship between mortality and moving average of mean temperature for lag 0–21 days/relative humidity for lag 0–7 days was controlled. The R code and examples of data are provided in the Supplementary materials (available here). The same results are observed for conditional Poisson regression model and conditional logistic regression model (Table 1). If the count data depart from Poisson distribution, the quasi-Poisson function can be applied to accommodate overdispersion. 

**Table 1:** The association between mortality and DTR derived from different models and parameter settings.

Model	Function	Argument: method	Relative risk	95% confidence interval	
				Lower	Upper
Conditional Poisson regression	gnm()	Poisson	1.000993	1.000637	1.001349
		Quasi-Poisson	1.000993	1.000574	1.001412
Conditional logistic regression	clogit()	“Breslow”	1.000993	1.000637	1.001349
		“Approximate”	1.000993	1.000637	1.001349

In summary, we show how to use space-time-stratified case-crossover design for multilocation time-series data to assess the risks of health from environmental exposure. Space-time-stratified case-crossover design is easy to be applied by one-stage analysis. It could provide reliable effect estimates through matching cases and controls to control for spatial variation, long-term trend, and seasonality. Moreover, alternative statistical methods applicable to space-time-stratified case-crossover design further enable researchers to conduct analyses with various types of data formats. The decision about which method to choose depends on the exposure data. If there are only community-level (e.g., city-level) exposure data, both methods will provide the same effect estimates. If there are individual exposure data, individual-time-stratified case-crossover design performed by conditional logistic regression is recommended, because aggregating individual into daily counts and average individual exposure to community level will introduce exposure bias. Nevertheless, simulation studies are still warranted to explore the performance of different statistical models and their applicability.

## Supplementary Materials

Supplementary Materials Supplementary material 1 1. Table S1: an example of time-series data applicable for conditional Poisson regression. 2. Table S2: an example of matched case-control data applicable for conditional Logistic regression. Supplementary material 2 1: R codes.
