# Supplementary material for: Space-Time-Stratified Case-Crossover Design in Environmental Epidemiology Study
Source: Health Data Sci. 2021 Oct 7;2021:9870798. doi: 10.34133/2021/9870798 (PMC10880144; doi:10.34133/2021/9870798)
Supplement: Supplementary Materials — Supplementary material 1 1. Table S1: an example of time-series data applicable for conditional Poisson regression. 2. Table S2: an example of matched case-control data applicable for conditional Logistic regression. Supplementary material 2 1: R codes. [file 9870798.f1.docx]

**Supplementary materials**

**Space-time-stratified case-crossover design in environmental epidemiology study**

**Table of contents**

**Supplementary material 1**

**1. Table S1**. An example of time-series data applicable for conditional Poisson regression.

**2. Table S2**. An example of matched case-control data applicable for conditional Logistic regression.

**Supplementary material 2**

**1. R codes**

**Supplementary material 1**

The data is available at the following link:

<https://github.com/yaowu-ops/space-time-stratifed-case-crossover/tree/main/data>.

**Table S1**. An example of time-series data applicable for conditional Poisson regression.

| Date | Region | Death | DTR | Stratum |
| --- | --- | --- | --- | --- |
| 01/01/1993 | York&Hum | 208 | 5.20 | York&Hum-1993-January-Fri |
| 02/01/1993 | York&Hum | 192 | 3.89 | York&Hum-1993-January-Sat |
| 03/01/1993 | York&Hum | 181 | 7.26 | York&Hum-1993-January-Sun |
| 04/01/1993 | York&Hum | 228 | 10.52 | York&Hum-1993-January-Mon |
| 05/01/1993 | York&Hum | 165 | 9.21 | York&Hum-1993-January-Tue |
| 06/01/1993 | York&Hum | 188 | 2.74 | York&Hum-1993-January-Wed |
| 07/01/1993 | York&Hum | 207 | 6.43 | York&Hum-1993-January-Thu |
| 08/01/1993 | York&Hum | 172 | 6.91 | York&Hum-1993-January-Fri |
| 09/01/1993 | York&Hum | 203 | 7.30 | York&Hum-1993-January-Sat |
| 10/01/1993 | York&Hum | 211 | 7.23 | York&Hum-1993-January-Sun |

**Table S2**. An example of matched case-control data applicable for conditional Logistic regression.

| Status | Stratum | Weights | DTR | Date | Region |
| --- | --- | --- | --- | --- | --- |
| 1 | 1 | 208 | 5.20 | 01/01/1993 | York&Hum |
| 0 | 1 | 208 | 6.91 | 08/01/1993 | York&Hum |
| 0 | 1 | 208 | 8.99 | 15/01/1993 | York&Hum |
| 0 | 1 | 208 | 4.54 | 22/01/1993 | York&Hum |
| 0 | 1 | 208 | 2.17 | 29/01/1993 | York&Hum |
| 1 | 2 | 192 | 3.89 | 02/01/1993 | York&Hum |
| 0 | 2 | 192 | 7.30 | 09/01/1993 | York&Hum |
| 0 | 2 | 192 | 8.08 | 16/01/1993 | York&Hum |
| 0 | 2 | 192 | 9.42 | 23/01/1993 | York&Hum |
| 0 | 2 | 192 | 4.27 | 30/01/1993 | York&Hum |

Note: variable “Weights” denotes the death count of each day.

**Supplementary material 2**

**R codes**

library("dlnm");library(splines);library(gnm);library(survival)

####################################################################################

# FIT A CONDITIONAL POISSON MODEL WITH A REGION-SPECIFIC YEAR X MONTH X DOW STRATUM

# IMPORT DATA

load("data_poisson.RData")

# RUN THE MODEL AND OBTAIN PREDICTIONS

model_cpoisson <- gnm(death~dtr+ns(templag0_21,4)+ns(rhlag0_7,3), data=data_poisson,

family=poisson(),

eliminate=factor(stratum))

summary(model_cpoisson)

# RUN THE MODEL AND OBTAIN PREDICTIONS (ALLOW FOR OVERDISPERSION)

model_cquasipoisson <- gnm(death~dtr+ns(templag0_21,4)+ns(rhlag0_7,3), data=data_poisson,

family=quasipoisson(),

eliminate=factor(stratum))

summary(model_cquasipoisson)

####################################################################################

# FIT A CONDITIONAL LOGISTIC MODEL WITH A REGION-SPECIFIC YEAR X MONTH X DOW STRATUM

# FORMAT: ID-MATCHED CASE-CONTROL DATA

# IMPORT DATA

load("data_clogit.RData")

# PERFORM CONDITIONAL LOGISTIC MODEL USING CLOGIT

# NOTE: THE DEFAULT METHOD DOES NOT WORK IN CLOGIT, RESEARCHERS SHOULD SPECIFY ONE METHOD.

# BRESLOW METHOD CONSIDERS EACH OF THE EVENTS AT A GIVEN TIME AS DISTINCT FROM EACH OTHER AND ALLOWS ALL FAILED SUBJECTS TO CONTRIBUTE WITH THE SAME WEIGHT TO THE RISK SET.

# (FROM: INTRODUCTION TO SURVIVAL ANALYSIS IN PRACTISE (2019) MACHINE LEARNING AND KNOWLEDGE EXTRACTION

model_clogit1 <- clogit(status ~ dtr+ns(templag0_21,4)+ns(rhlag0_7,3)+strata(stratum),

weights=weights,

method = "breslow",data=data_clogit)

summary(model_clogit1)

model_clogit2 <- clogit(status ~ dtr+ns(templag0_21,4)+ns(rhlag0_7,3)+strata(stratum),

weights=weights,

method = "approximate",data=data_clogit)

summary(model_clogit2)
